# Errata

**Published:** 1781-06

**Authors:** 


					p. 285, 1, 6, for eftett read aftect.?

				

